# Appropriate Surgical Repair of Ventricular Free Wall Rupture after Infarction: a Case Report

**DOI:** 10.21470/1678-9741-2018-0205

**Published:** 2019

**Authors:** Umut Serhat Sanrı, Kadir Kaan Özsin, Faruk Toktaş, Şenol Yavuz

**Affiliations:** 1 Department of Cardiovascular Surgery, University of Health Sciences, Bursa Yüksek İhtisas Research and Trainning Hospital, Bursa, Turkey.

**Keywords:** Heart Rupture, Myocardial Infarction - Surgery

## Abstract

Ventricular free wall rupture is a fatal mechanical complication of acute myocardial infarction. In some cases it can be represented as subacute clinic and may not cause death in a few minutes. Acute pseudo-aneurysms are extremely unstable and bound to fatal rupture. Herein we report a male patient who suffered dyspnea and mild chest pain, 4 weeks after acute ST-segment elevation myocardial infarction.

**Table:** 

Abbreviations, acronyms & symbols	
AMI	= Acute myocardial infarction
CPB	= Cardiopulmonary bypass
ECG	= Electrocardiogram
LVEF	= Left ventricular ejection fraction
LVFWR	= Left ventricular free wall rupture
MI	= Myocardial infarction
STEMI	= ST-segment elevation myocardial infarction
VFWR	= Ventricular free wall rupture

## INTRODUCTION

Ventricular free wall rupture (VFWR) is a rare but catastrophic and fatal mechanical complication of acute myocardial infarction (AMI). This clinical situation may be responsible for 14% to 26% of total early deaths in patients with AMI^[1]^. In some cases it can be considered as subacute clinic and not be typical of an acute rupture that may cause death fairly quickly. Acute pseudo-aneurysms, a variant of myocardial ruptures, are extremely unstable and bound to fatal rupture. Chronic pseudo-aneurysms are usually detected because of symptoms, less often incidentally. Pseudo-aneurysms develop when cardiac rupture is contained by pericardial adhesions^[2]^.

We present a male patient who suffered dyspnea and mild chest pain, 4 weeks after acute ST-segment elevation myocardial infarction (STEMI).

## CASE REPORT

**Fig. 1:**
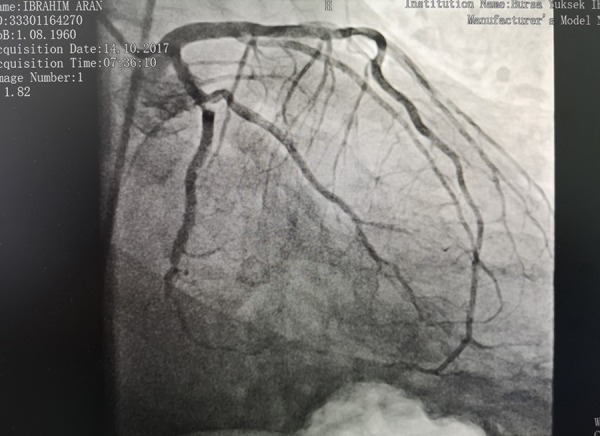
Coronary angiography image shows the Cx lesion.

**Fig. 2:**
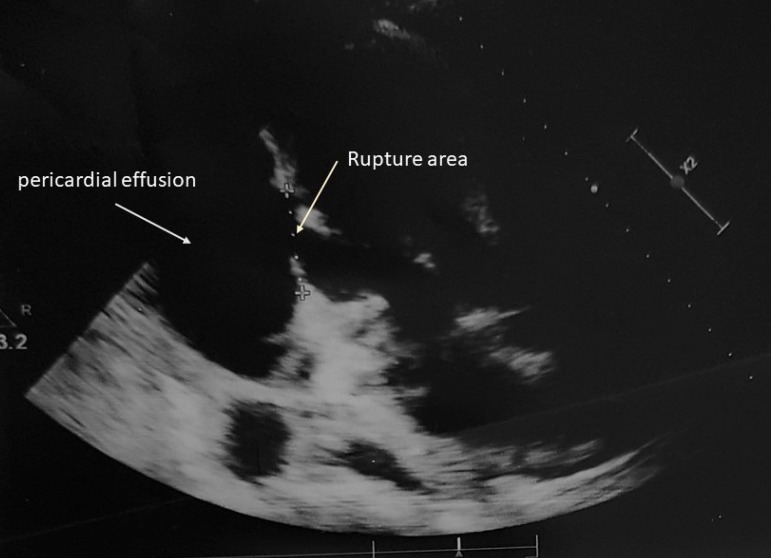
Echocardiogram showing the myocardial rupture area and pericardial effusion.

**Fig. 3:**
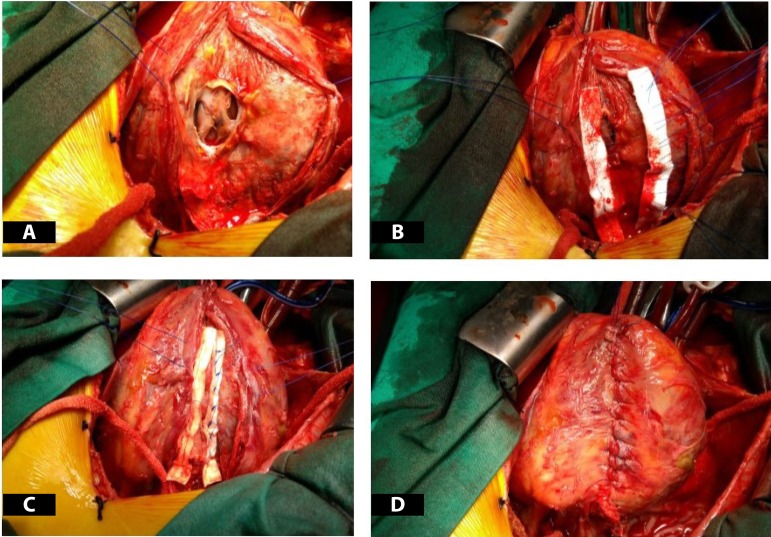
Intra-operative photos showing free wall rupture and repair; A)apperance of free wall rupture, B and C)repair of free wall rupture with teflon felt, D)repaired free wall rupture.

**Fig. 4:**
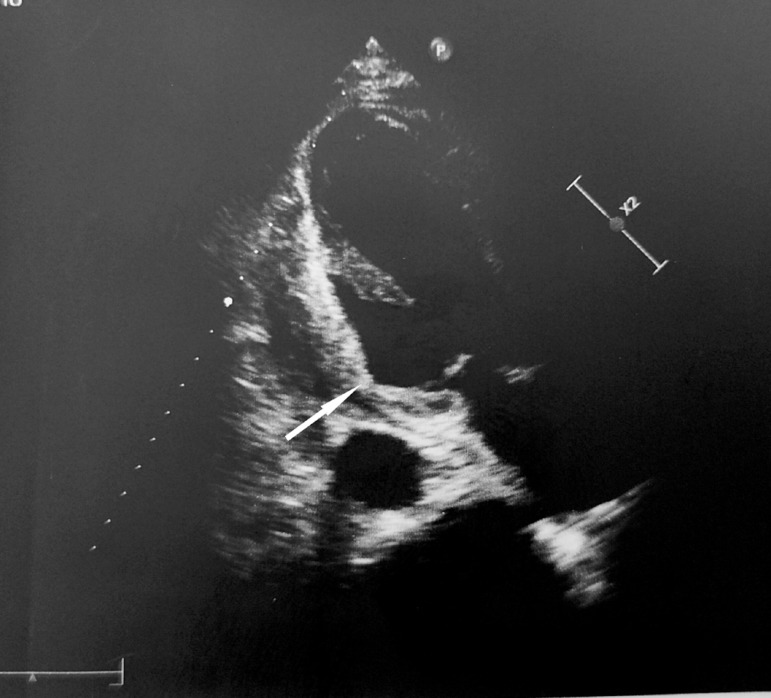
Arrow shows closed myocardial defect.

A 57-year-old male was admitted to our hospital with a 5-day history of severe dizziness, mild chest pain and dyspnea. On admission, he had a pulse rate of 115 bpm, a blood pressure of 80/60 mmHg, and a respiratory rate of 24. He had a history of hypertension and no diabetes mellitus. On physical examination, there were no pathological signs for heart sounds, but some wet rales were detected in the lower lung region. There was minimal bilateral leg edema. Cardiac troponin T levels were clearly normal. The electrocardiogram (ECG) did not show any new myocardial infarction (MI) sign. When checking patient history, we discovered that he had suffered STEMI four weeks prior. In the coronary angiography performed, an occlusive lesion was detected in the distal circumflex artery and he was discharged with medical treatment (Figure 1). There was a right type of coronary dominance. Transthoracic echocardiography showed a 3-cm wide wall defect on the posterior side of the left ventricle, a 57-mm pericardial effusion and the left ventricular ejection fraction (LVEF) was 40% (Figure 2). The operation was performed immediately via standard median sternotomy. Adherence of the pericardium was at an advanced level. The anterior part of the pericardium was dissected carefully for cannulation. We used cold potassium-based cardioplegia (Plegisol®, Baxter, Chicago, USA) for cardiac arrest and continued with cold blood cardioplegia. After a standard cardiopulmonary bypass (CPB) procedure, we separated the entire pericardium from the heart. On the posterior region we detected a formation like pseudo-aneurysm which was a 2-mm thick membrane formed by adhesion. It was almost completely covered the posterior face of the left ventricle. After opening this membrane, the myocardial defect was seen (Figure 3A). We considered that it was a subacute situation because of the non-existence of fragile necrotic tissue. The defect was sutured with eight separated and teflon felt supported 3-0 prolene sutures (Figure 3B and C). Tissue glue was applied on the sutured line. Finally, the residual capsule existent from adhesion was closed with a 3-0 prolene continuously (Figure 3D). There was no need for a concomitant CABG according to angiography and intraoperative examination. No bleeding occurred from suture line or anywhere else. Weaning from CPB was performed with the support of an intra-aortic balloon pump and dopamine medication. While aortic cross clamp time was eighty-seven minutes, total CPB time was one hundred and twenty one minutes. Postoperative transthoracic echocardiography showed normal left and right ventricular size (LVEF of 45%) without pseudo-aneurysm and minimal pericardial effusion (Figure 4). Intra-aortic balloon pump was removed on the second postoperative day. He was discharged without any follow up events at postoperative day seven from our hospital.

## DISCUSSION

Left ventricular free wall rupture is the one of the severe mechanical complications of the MI. In a large transmural AMI patient without a history of angina or prior MI, there is an increased risk of rupture because there is no collateral circulation present. In our case, percutaneous coronary intervention was not performed on the occlusion of the distal part of the main body of the circumflex artery, and the angiogram showed no collateral circulation in that region. Adherent thrombus or pericardial adhesions may help a few patients to survive. Left ventricular wall ruptures and pseudoaneurysms are usually inclined to originate from the posterior basal and rarely at the apical segment of the heart after occlusion of the related coronary artery, as in our case. A postinfarction true aneurysm is caused by scar formation. True aneurysm occur at apical segment of left ventricle, consequent to occlusion of the left anterior descending artery^[2]^.

Free wall rupture usually occurs within the first week after infarction. However, the median time for diagnosis is typically four months after the infarct. Symptoms and signs of acute ruptures are typically presenting as chest pain, syncope, cyanosis, hypotension, bradycardia, pulsus paradoxus, elevated venous pressures, very feeble heart sounds, electromechanical dissociation and cardiogenic shock^[3]^. Pseudoaneurysm in subacute clinical manifestations can be diagnosed as incidental because it has not specific clinical findings. Our case admitted to the hospital with chest pain and shortness of breath which started a week prior, similar to the subacute phase and he had suffered STEMI four weeks prior. The diagnosis can be made with suspicion about mechanical complications due to the presence of related symptoms in a patient with MI. Transthoracic echocardiography is usually sufficient for diagnosis. Echocardiography may show echogenicity of the pericardial thrombus, regional akinetic and thinned myocardial tissue, and ruptured wall with colored doppler. In addition, presence of a narrow neck in Color Doppler Echocardiography suggests the pseudoaneurysm. In our case, there was no classic pseudoaneurysm image on echocardiography. A wide rupture, bordered by pericardial tissue, was detected in the postero-basal wall of the left ventricle which has flow within and no hematoma. Due to a hemodynamic instability, the role of pre-operative coronary angiography in this group of patients remains controversial. In addition, If the patient is stable, then cardiac computed tomography and magnetic resonance imaging could be considered.

There are many types of treatment approach for LVFWR. Nasir et al.^[4]^reported that surgical intervention is superior to conservative treatment in the treatment of free wall rupture. One of them is the surgical approach, including prosthetic patch replacement after infarctectomy or closure with direct mattress sutures under CPB^[2]^. Previous studies report excellent results with the sutureless technique by which a patch of Teflon and dacron was glued to the pericardium^[5]^. In this procedure, fibrin glue-containing (TachoComb^®^ and TachoSil^®^) patches are applied to the ruptured area and compression is enforced on the oozing surface for complete hemostasis. Misawa reported this procedure without CPB in a review^[6]^. But in this review, thirty-three cases out of 35 were oozing type and just two of them were blowout type ruptures. In our case, LVFWR was not oozing type but blowout, thus we did not consider sutureless technique as a first choice. Our case was in chronical stage, therefore we performed primary repair with separated prolene suture and teflon felt. In our case we accept that persistent occlusion of infarct-related artery responsible for LVFWR. Interestingly in our case, after dyspnea and chest pain started again, he insisted on not being taken to the hospital. He spent one week without treatment at his home. The chance of the patient was pericardial adhesions. Although postinfarct complications may occur even if adequate intervention is performed for coronary artery lesion, we think that the rupture in our case is caused by the absence of percutaneous coronary intervention in the occlusion of the distal portion of the circumflex artery. We believe that we applied the most appropriate surgical treatment for our patient. 

## CONCLUSION

In conclusion, we suggest close follow-up for patients with AMI which treated inadequately. In addition, surgical techniques should be considered according to the rupture type and time after AMI. 

**Table:** 

Authors' roles & responsibilities	
USS	Substantial contributions to the conception or design of the work; or the acquisition, analysis, or interpretation of data for the work; final approval of the version to be published
KKO	Substantial contributions to the conception or design of the work; or the acquisition, analysis, or interpretation of data for the work; final approval of the version to be published
FT	Drafting the work or revising it critically for important intellectual content; final approval of the version to be published
ŞY	Drafting the work or revising it critically for important intellectual content; final approval of the version to be published
